# P073: Outbreak of carbapenemase-producing Pseudomonas aeruginosa in a tertiary care hospital

**DOI:** 10.1186/2047-2994-2-S1-P73

**Published:** 2013-06-20

**Authors:** P Navarro, M Cantero Caballero, I Wijers, I Cerón, E Martinez de Albeniz, P Rodriguez

**Affiliations:** 1 Department of Preventive Medicine and Quality Management, General University Hospital Gregorio Marañón, Madrid, Spain

## Introduction

Carbapenemase-mediated resistance to carbapenems in *Pseudomonas Aeruginosa* has increased in Spain since 2003.

## Objectives

To describe an outbreak of multi-resistant carbapenemase-producing *Pseudomonas Aeruginosa*, in a bone marrow transplant unit (BMTU) of a tertiary care hospital.

## Methods

Descriptive study of the outbreak and the control measures implemented. Surveillance cultures from patients staying in the BMTU were taken in order to detect colonization, as well as environmental samples.

## Results

In March 2012 a carbapenemase-producing *P. Aeruginosa* isolate resistant to carbapenems and beta-lactam antibiotics was isolated from a wound culture of a patient admitted to our BMTU. The patient had previously presented a sepsis secondary to ecthyma gangrenosum caused by carbapenem-resistant but non-carbapenemase-producing *P. Aeruginosa* and had prolonged broad-spectrum antibiotic therapy. Carbapenemase-producing *P. Aeruginosa* was isolated from all of the following cultures. During the month of April 2012 two new cases were identified on the BMTU, both suffering from symptomatic urinary tract infections, with detection of *P. Aeruginosa* in urine cultures. PCR was used to confirm that it was the same VIM-type strain in all of the three cases. Control measures included: contact isolation in individual rooms, specialized personnel attending the isolated patients, enhanced standard precautions, additional cleaning of patient rooms and enhanced cleaning and disinfection of medical materials. In June 2012, two of the three patients were discharged and one of them died from an unrelated cause. No new cases have been detected on the BMTU.

## Conclusion

The capacity of microorganisms, and especially *P. Aeruginosa*, to acquire new mechanisms of resistance under antibiotic selection pressure, poses important therapeutic problems and difficulties for the control of healthcare-associated infections.

## Disclosure of interest

None declared

